# Multidrug Resistant *Acinetobacter*

**DOI:** 10.4103/0974-777X.68538

**Published:** 2010

**Authors:** Vikas Manchanda, Sinha Sanchaita, NP Singh

**Affiliations:** *Clinical Microbiology and Infectious Diseases Division, Chacha Nehru Bal Chikitsalaya and associated Maulana Azad Medical College, Government of NCT of Delhi, Geeta Colony, Delhi – 110031, India*; 1,2 *Department of Microbiology, University College of Medical Sciences and Guru Teg Bahadur Hospital, Dilshad Garden, Delhi - 110095, India*

**Keywords:** *Acinetobacter*, Antimicrobial resistance, Antimicrobial therapy, Clinical implications, Hospital acquired infections, Nosocomial infections, Outbreak, Infection control, Antimicrobial stewardship

## Abstract

Emergence and spread of *Acinetobacter* species, resistant to most of the available antimicrobial agents, is an area of great concern. It is now being frequently associated with healthcare associated infections. Literature was searched at PUBMED, Google Scholar, and Cochrane Library, using the terms ‘*Acinetobacter* Resistance, multidrug resistant (MDR), Antimicrobial Therapy, Outbreak, Colistin, Tigecycline, AmpC enzymes, and carbapenemases in various combinations. The terms such as MDR, Extensively Drug Resistant (XDR), and Pan Drug Resistant (PDR) have been used in published literature with varied definitions, leading to confusion in the correlation of data from various studies. In this review various mechanisms of resistance in the *Acinetobacter* species have been discussed. The review also probes upon the current therapeutic options, including combination therapies available to treat infections due to resistant *Acinetobacter* species in adults as well as children. There is an urgent need to enforce infection control measures and antimicrobial stewardship programs to prevent the further spread of these resistant *Acinetobacter* species and to delay the emergence of increased resistance in the bacteria.

## INTRODUCTION

Management of multidrug-resistant *Acinetobacter* spp. infections is a great challenge for physicians and clinical microbiologists. Its ability to survive in a hospital milieu and its ability to persist for extended periods of time on surfaces makes it a frequent cause for healthcare-associated infections and it has led to multiple outbreaks.[1,2] It causes a wide spectrum of infections that include pneumonia, bacteremia, meningitis, urinary tract infection, and wound infection.

Although, Beijerinck (1911), a Dutch microbiologist, isolated the organism from the soil by enrichment in calcium acetate containing minimal medium, and named it *Micrococcus calcoaceticus*, genus *Acinetobacter* was not definitively established until 1971.[3] On the basis of the DNA relatedness criteria, Bouvet and Grimont, in 1986, distinguished 12 DNA (hybridization) groups or genospecies, some of which were given formal species names, including *A. baumannii, A. calcoaceticus, A. haemolyticus, A. johnsonii, A. junii*, and *A. lwoffii*.[4] At present, more than 25 species of *Acinetobacter* have been recognized via DNA–DNA hybridization within the genus and seven have been given formal species names. Among these species, *A. calcoaceticus, A. baumannii, Acinetobacter* genomic species 3, and *Acinetobacter* genomic species 13TU, have an extremely close relationship and are difficult to distinguish from each other by phenotypic tests alone. Therefore, they have been grouped as the *A. calcoaceticus – A. baumannii* complex.[5,6] This group accounts for 80% of the clinical infections caused by *Acinetobacter* spp.[6–10]

## DEFINITIONS

Definitions of multidrug-resistant *Acinetobacter* species vary when referring to a wide array of genotypes and phenotypes.[11] Different terms like ‘multidrug resistant (MDR)’, ‘extensive drug resistant (XDR),’ and ‘pandrug resistant (PDR)’ have been used with varied definitions to describe the extent of antimicrobial resistance among *Acinetobacter* spp. However, to date, unlike *Mycobacterium tuberculosis*, internationally, there are no accepted definitions for the extent of resistance in the bacteria. Arbitrarily used terms have thus caused great confusion making it difficult for the available literature to be analyzed.[12]

**Figure 1:**
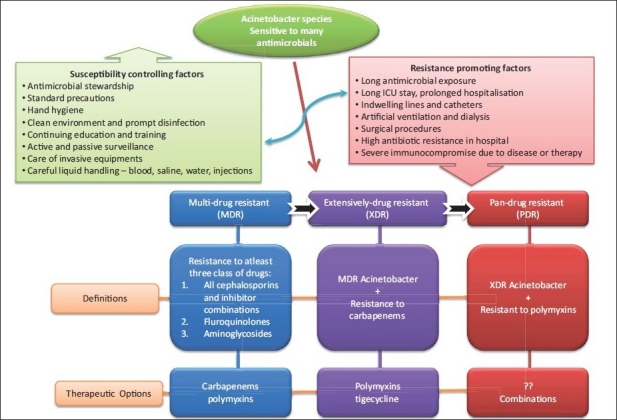
Definition of drug resistant *Acinetobacter* species along with therapeutic options. Resistance promoting factors and Suceptibility controlling factors has been summarised

In the current review ‘MDR *Acinetobacter* spp.’ shall be defined as the isolate resistant to at least three classes of antimicrobial agents — all penicillins and cephalosporins (including inhibitor combinations), fluroquinolones, and aminoglycosides. ‘XDR *Acinetobacter* spp.’ shall be the *Acinetobacter* spp. isolate that is resistant to the three classes of antimicrobials described above (MDR) and shall also be resistant to carbapenems. Finally, ‘PDR *Acinetobacter* spp.’ shall be the XDR *Acinetobacter* spp. that is resistant to polymyxins and tigecycline [Figure 1]. The above definitions have been described keeping in view the different mechanisms of resistance known till date and the antimicrobials being used to treat various *Acinetobacter* spp. infections. These definitions further help to clearly define the extent of resistance and rational antimicrobial therapy.

## HABITAT AND EPIDEMIOLOGY

Widely distributed in soil and water, *A. baumannii* grows at various temperatures and pH environments and uses a vast variety of substrates for its growth.[13,14] In nature, *Acinetobacter* is most commonly found in soil and water, but has also been isolated from animals. *Acinetobacter baumannii* normally inhabits human skin, mucous membranes, and soil. *A. calcoaceticus* is found in water and soil and on vegetables; *Acinetobacter* genomic species 3 is found in water and soil, on vegetables, and on human skin; *A. johnsonii* is found in water and soil, on human skin, and in human feces; *A. lwoffii* and *A. radioresistens* are found on human skin; and *Acinetobacter* genomic species 11 is found in water and soil, on vegetables, and in the human intestinal tract.[15] It has also been isolated from the human body lice of homeless people in France.[16]

In humans, *Acinetobacter* has been isolated from all culturable sites. *Acinetobacter* can form part of the bacterial flora of the skin, particularly in moist regions such as the axillae, groin, and toe webs, and up to 43% of healthy adults can have colonization of skin and mucous membranes, with higher rates among hospital personnel and patients.[17] The most frequently isolated species in this study includes *A. lwoffii* (58%), *A. johnsonii* (20%), *A. junii* (10%), and *Acinetobacter* genomic species 3 (6%).[17] In a similar study, a carrier rate of 44% was found in healthy volunteers, with *A. lwoffii* (61%), *Acinetobacter* genomic species 15BJ (12%), *A. radioresistens* (8%), and *Acinetobacter* genomic species 3 (5%) being the most prevalent species.[18] In another study of the fecal carriage of *Acinetobacter*, a carrier rate of 25% was observed among healthy individuals, with *A. johnsonii* and *Acinetobacter* genomic species 11 being the predominant species.[19] It has also been found occasionally in the oral cavity and respiratory tract of healthy adults, but the carriage rate of *Acinetobacter* spp. in non-hospitalized patients, apart from on the skin, is normally low.[7,20,21] Interestingly, *A. baumannii*, the most important nosocomial *Acinetobacter* sp., has been rarely found on human skin (0.5 and 3%) and in human feces (0.8%).[17–19]

Among the patients who are hospitalized in non-intensive care units, the skin carriage rate of *Acinetobacter* spp. has been found to be as high as 75%.[17] In particular, high colonization rates have been observed in Intensive Care Unit (ICU) patients, especially of the respiratory tract. Sources for colonization or infection with multidrug-resistant *Acinetobacter* species in hospitalized patients are summarized in Table 1. *Acinetobacter* is a hydrophilic organism and preferentially colonizes in aquatic environments. *Acinetobacter* spp. has been documented to survive in hospital environments. The reservoirs of this pathogen are poorly understood.[26] The organism can survive for long periods on both dry and moist surfaces.[26] Survival is probably helped by the ability of *Acinetobacter* spp. to grow at a range of different temperatures and pH values.[7,22,26,27] *Acinetobacter* spp. has commonly been isolated from the hospital environment and hospitalized patients.[7,22]

**Table 1:** Sources of colonization or infection with multidrug-resistant *Acinetobacter* species in a hospital environment

Hands of the hospital staff Respiratory therapy equipment Food (including hospital food) Tap water Infusion pumps Mattresses, pillows, bed curtains and blankets in vicinity of infected patients Soap dispensers Fomites like bed rails, stainless steel trolleys, door handles, telephone handles, tabletops Hospital sink traps Hospital floor

Patients with *Acinetobacter* colonization often have a history of prolonged hospitalization or antimicrobial therapy (with antibiotics that have little or no activity against *Acinetobacter*). Residency in an ICU, particularly in the presence of other patients who are colonized with *Acinetobacter*, predisposes patients to colonization. It is particularly seen in patients who are intubated and in those who have multiple intravenous lines, monitoring devices, surgical drains, or indwelling urinary catheters.[22–26] It is often cultured from hospitalized patient’s sputum or respiratory secretions, wounds, and urine, and commonly colonizes in irrigating solutions and intravenous fluids. *Acinetobacter* infections usually involve organ systems with a high fluid content (e.g., respiratory tract, blood, CSF, peritoneal fluid, urinary tract).[28–30] Invasive devices used to facilitate fluid monitoring, administer medications, and provide lifesaving support may also be sources of colonization.[31] This indicates the hardy nature of *Acinetobacter* spp., allowing it to survive in the environment for several days, even in dry conditions on particles and dust, thereby probably contributing to the development and persistence of outbreaks. Several studies have shown the capacity of this organism to survive on dry surfaces, for durations longer than that found for *Staphylococcus aureus*.[7,32]

Various risk factors for colonization or infection with multidrug-resistant *Acinetobacter* species are summarized in Table 2. Many case control studies have revealed that prior exposure to antimicrobial therapy has been the most common risk factor identified in multivariate analysis. Carbapenems and third-generation cephalosporins are the most commonly implicated antibiotics, followed by fluoroquinolones, aminoglycosides, and metronidazole. The second most common risk factor identified in case-control studies is mechanical ventilation.[35] Other risk factors include a stay in an ICU, length of ICU and hospital stay, severity of the illness, recent surgery, and invasive procedures.[35–39] Furthermore, studies on *A. baumannii* outbreaks have revealed environmental contamination as an important risk factor in the causation of outbreaks.

**Table 2:** Risk factors for colonization or infection with multidrug-resistant *Acinetobacter* species.[22–27]

Prolonged length of hospital stay Exposure to an intensive care unit (ICU) Receipt of mechanical ventilation Colonization pressure Exposure to antimicrobial agents esp., carbapenems, colistin Recent surgery Invasive procedures Underlying severity of illness

In a recent matched case-control study undertaken to evaluate risk factors associated with the isolation of colistin-resistant *A. baumannii* the only independent risk factor identified in the multivariate analysis was the previous use of colistin.[40]

Multivariable analysis controlling for severity of illness and underlying disease identified an independent association between patients with MDR *Acinetobacter* infection and increased length of stay in the hospital and intensive care unit compared to patients with susceptible *Acinetobacter* infection (odds ratio [OR] 2.5, 95% confidence interval [CI] 1.2 – 5.2 and OR 2.1, 95% CI 1.0 – 4.3, respectively) and uninfected patients (OR 2.5, 95% CI 1.2 – 5.4 and OR 4.2, 95% CI 1.5 – 11.6, respectively).[41]

During the outbreaks, extensive contamination of the environment, including respirators and air samplers in the vicinity of the infected or colonized patients have been documented. Bed linen of colonized patients is consistently culture positive for *Acinetobacter* species, whereas, the bed linen of non-colonized patients is found to habour *Acinetobacter* spp. on several occasions. It has also been recovered from mattresses, pillows, bed curtains, and blankets in the immediate vicinity of infected patients. It has also been isolated from food (including hospital food), ventilator equipment, suctioning equipment, infusion pumps, stainless steel trolleys, pillows, mattresses, tap water, bed rails, humidifiers, soap dispensers, and other sources. Also, other fomites like door handles, telephone handles, tabletops, and so on have tested positive for *Acinetobacter* species during outbreaks, probably contaminated by the hands of the staff. One or more epidemic *Acinetobacter* species clones often coexist with the endemic strains, making it difficult to detect and control transmission.[42,43]

Compounding to the problem of the ease to survive in a hospital environment and increasing antibiotic resistance, is the ability of this organism to form biofilms. It has been shown that *Acinetobacter* species can form biofilms on the surface of various implants and also in the environment. In such situations, the antibiotics for which it is showing *in vitro* susceptibility will also be ineffective in treating the infection.[44,45]

## MECHANISMS OF RESISTANCE TO ANTIMICROBIAL AGENTS

During the early 1970s the clinical isolates of *Acinetobacter* spp. were usually susceptible to gentamicin, minocycline, nalidixic acid, ampicillin, or carbenicillin, singly or in a combination therapy. However, since 1975, increasing resistance started appearing in almost all groups of drugs including the first and second generation cephalosporins. Initially they retained at least partial susceptibility against the third and fourth generation cephalosporins, fluoroquinolones, semi synthetic aminoglycosides, and carbapenems, with almost 100% isolates retaining susceptibility to imipenem. However, during late 1980s and 1990s, worldwide emergence and spread of *Acinetobacter* strains resistant to imipenem further limited the therapeutic alternatives.[1,7,26,46–48] By the late 1990s, carbapenems were the only useful agents remaining that could combat many severe *Acinetobacter* infections. Furthermore, due to the emergence of carbapenem resistance in the strains of *A. baumannii*, largely through a clonal spread, the therapeutic options are decreasing.[49–51] Multiple mechanisms have been found to be responsible for the resistance to carbapenems in *A. baumannii*.

The mechanisms of antimicrobial resistance in *A. baumannii* generally falls into three broad categories: (1) antimicrobial-inactivating enzymes, (2) reduced access to bacterial targets (due to decreased outer membrane permeability caused by the loss or reduced expression of porins, overexpression of multidrug efflux pumps) and (3) mutations that change targets or cellular functions (alterations in penicillin-binding proteins; PBPs).[51,52] A combination of several mechanisms may be present in the same microorganism, as has also been observed in other gram-negative bacteria.[51] Different mechanisms of resistance in the *Acinetobacter* species are summarized in Table 3.

**Table 3:** Mechanisms of antibiotic resistance found in *Acinetobacter* species^1^,^7^,^54^,^55^

Mechanism of resistance	Genetic mechanisms	Antimicrobials affected
A. Antimicrobial inactivating (hydrolysing) enzymes		
Amp C Beta-lactamases [*Acinetobacter*-derived cephalosporinases (ADCs)]	Chromosomal mediated Insertion sequences ISAba1 and IS1135 increase production of beta-lactamase	Extended spectrum cephalosporins (including 3^rd^ generation and cephamycin group); cefepime and carbapenems are spared
Ambler class D OXA-type enzymes	Chromosomal and Plasmid mediated	Carbapenems
Ambler class B metallo–b-lactamases (MBLs), such as VIM and IMP	Mobile genetic elements	Carbapenems
Ambler class A ESBLs (TEM, SHV)	Plasmid, chromosomal or mobile genetic elements	All cephalosporins (including 3^rd^ generation) except cephamycin group
B. Reduced access to bacterial targets		
Altered porin channels and other outer membrane proteins	Point mutations	Carbapenems
C. Mutations that change targets or cellular functions		
DNA topoisomerase mutations	Point mutations in the bacterial targets gyrA and parC topoisomerase enzymes	Quinolones
Aminoglycoside-modifying enzyme	Plasmid, transposons	Aminoglycosides
Production of efflux pumps	Point mutations	Tigecycline, aminoglycosides, quinolones, tetracyclines
Modification of cell membrane lipopolysccarides	Point mutations	Colistin

### Antimicrobial inactivating enzymes

*Acinetobacter* species possess a wide array of beta-lactamases that hydrolyze and confer resistance to penicillins, cephalosporins, and carbapenems. *A. baumannii* inherently produces an AmpC-type cephalosporinase also known as *Acinetobacter*-derived cephalosporinases (ADCs). These enzymes, when expressed at a basal level, do not reduce the efficacy of expanded spectrum cephalosporins.[15,53–57] ADCs hydrolyze amino-penicillins and extended spectrum cephalosporins. Unlike that of AmpC enzymes found in other gram-negative organisms, inducible AmpC expression does not occur in *A. baumannii*.[57,58] The key determinant regulating overexpression of this enzyme in *A. baumannii* is the presence of an upstream insertion sequence (IS) element known as ISAba1, which provides an efficient promoter.[56,58–60] ISAba1 is widespread in *A. baumannii*, with up to 13 copies per cell. The presence of this element correlates very well with the increased AmpC gene expression and resistance to extended-spectrum cephalosporins.[15] Cefepime and carbapenems appear to be resistant to the hydrolysis caused by these enzymes.[53]

The main cause of carbapenem resistance in *A. baumannii* is class D (OXA) carbapenemases — another naturally occurring beta-lactamase in *A. baumannii* (OXA-51/66 group). Again, at their basal level of expressions, OXA-51-like enzymes are expressed poorly in most strains, which explains a low impact on susceptibilities to all beta-lactams including carbapenems. Expression of these enzymes also require (similar to ADCs) the insertion of ISAba1, upstream of the structural gene. The expression then leads to carbapenem resistance in *A. baumannii*.[52] Since the first description of a serine carbapenemase in *A. baumannii*, ARI-1 (OXA-23), in a clinical isolate from a blood culture in Scotland, in 1985, several variants of these enzymes have been reported globally, which include Scotland, Spain, France, Japan, Singapore, China, Brazil, Cuba, and Kuwait.[62–64] On the basis of sequence homology alone OXA carbapenemases can be divided into the following clusters: OXA-23-like (includes OXA-27 and OXA-49), OXA-(24)-40-like (includes OXA-25, OXA-26, and OXA-40), and OXA-58.[65–67] blaOXA-23 can be both chromosomal as well as plasmid-mediated and almost without exception is found in *A. baumannii*. OXA-23-like enzymes have been found repeatedly in the species from 1985 onwards, including outbreak strains collected in the UK, East Asia, and South America. It is present in one multi-resistant clone that is now prevalent in UK (OXA-23 clone1).[68] The OXA-24 group can also be encoded through either chromosomal or plasmid-mediated genes, although they appear less widespread than OXA-23, with reports generally restricted to Europe and the United States.[69]

OXA-58-like enzymes were first described recently in isolates from France, but were subsequently recognized as having occurred worldwide over the preceding eight to ten years.[68]

Unlike class A and B carbapenemases (e.g., KPC, VIM, IMP), OXA enzymes have low carbapenemase activity expressed *in vitro*, however, laboratory transfer and deletion experiments confirm their role in resistance.[70] This indicates that OXA enzymes might be more active in the bacterial periplasm because these enzymes can convert between the monomeric (less active) and dimeric (more active) forms, with the latter favored at high enzyme concentrations present in the periplasm.[71] Moreover, few isolates with OXA carbapenemases could have additional co-determinants of resistance, for example, lack of outer-membrane proteins or altered porins.[71]

Some *Acinetobacter* strains express Ambler class B metallo–beta-lactamases (MBLs), such as IMP, VIM, and SIM-1. These enzymes have been identified in *A. baumannii*. They confer a high level of resistance to carbapenems and to other beta-lactams except Aztreonam.[33] IMP-1 has been identified in Italy, South Korea, Japan, IMP-2 in Italy and Japan, IMP-4 in Hong Kong, IMP-5 in Portugal, and IMP-6 in Brazil. IMP-4 has also been identified in the *A. junii* isolate from Australia. VIM-1 has only been identified in Greece and VIM-2 beta-lactamases have been detected in *A. baumannii* isolates from South Korea.[61] The blaVIM-2 gene is located on two newly described integrons (class I integrons In105 and In106).[61] SIM-1 has been reported from *A. baumannii* in South Korea. MBLs pose a significant risk of spread as they are often located on mobile genetic elements that can be easily transferred among bacteria.[33,61] The genetic environment, classification, biochemistry of metallo-beta-lactamases, and their association with class 1 integrons that are part of transposons has been reviewed by Walsh *et al*.[72]

Extended-spectrum beta-lactamases (ESBLs) from the Ambler class A group have also been described for *A. baumannii*, but assessment of their true prevalence is hindered by difficulties with laboratory detection, especially in the presence of an AmpC. In *A. baumannii*, PER-1 was the first ESBL to be reported.[73] Initially this gene was confined to Turkey, but later has been identified globally including South Korea, Hungary, Italy, France, Belgium, Romania, United States, and China.[15] blaPER-1 is either plasmid or chromosomally encoded and also has an upstream IS element (ISPa12) that may enhance its expression.[74] Also, PER-2, has been identified and reported from Argentina.[15]

VEB-1 ESBL has also been identified in *A. baumannii*. The bla_VEB-1_ was identified as a form of gene cassette in class 1 integrons yet encoded on the chromosome.[75] This integron was identical to that identified in Pseudomonas aeruginosa in Thailand and was also associated with an upstream IS element (IS26), indicating the possible origin and mechanism of spread to *A. baumannii*.[75,76] VEB-1, has disseminated throughout hospitals in France (clonal dissemination) and has also been reported from Belgium and Argentina (VEB-1a).

ESBLs identified in *A. baumannii* include TEM-1, TEM-2, and the carbenicillinase CARB-5. The first two are narrow spectrum penicillinases, whereas, CARB-5 confers high level resistance to aminopenicillins and carbenicillins. The current clinical significance of these ESBLs is limited given the potency of other resistance determinants. A related enzyme SCO-1 has also been identified in *A. baumannii, A. junnii, A. johnsonii*, and *A. baylyi*. Moreover, TEM-92 and -116 have been identified in *A. baumannii* isolated from Italy and Netherlands, respectively, and SHV-12 from China and Netherlands.[77] Also, CTX-M-2 and CTX-M-43 have been described from Japan and Bolivia, respectively.[15,77] To date, Ambler class A carbapenemases (KPC, GES, SME, NMC, and IMI) have not been described for *A. baumannii*.[77]

### Porin channels and other outer membrane proteins

Porin channels and outer membrane proteins(OMPs) are important for the transport of antimicrobial agents into the cell, to gain access to bacterial targets. Carbapenem resistance in *Acinetobacter* species has been linked to the loss of proteins thought to be through porin channels from the outer membrane.[33] It is likely that beta-lactamases and outer-membrane alterations work together to confer resistance to beta-lactam agents.[61]

### Mutations that change targets or cellular functions

These resistance mechanisms involve point mutations that alter bacterial targets or functions, decreasing the affinity for antimicrobial agents or upregulating cellular functions, such as, the production of efflux pumps or other proteins. By reduction of transport into the periplasmic space via changes in porins or OMPs, the access to penicillin-binding proteins is reduced. With less beta-lactam entering the periplasmic space, the weak enzymatic activity of the beta-lactamase is amplified. Many outbreaks of infection with imipenem-resistant *A. baumannii* are due to porin loss. Various examples of the reduced number of porin channels and poor expression of genes resulting in porin loss or efficacy have been described in the review by Bonomo and Szabo.[61]

The role of efflux is to remove substances that could potentially disrupt the cytoplasmic membrane; however, from the point of view of antimicrobial resistance, efflux pumps have a potent ability to actively export beta-lactams, quinolones, and sometimes even aminoglycosides from cell cytoplasm. *Acinetobacter* species possess efflux pumps that are capable of actively removing a broad range of antimicrobial agents from the bacterial cell [Table 3].[61]

Besides resistance to the beta-lactam group of antimicrobials, resistance to other classes of antibiotics is almost always present in the *Acinetobacter* species. Aminoglycoside resistance is mediated by plasmid or transposons-coded Aminoglycoside-Modifying Enzymes (AMEs). Resistance due to all three types of AMEs — the acetylating, adenylating, and phosphorylating AMEs — have been identified in *A. baumannii*.[78,79]

Resistance to colistin is thought to be mediated with modifications of the lipopolysaccharides of the bacterial cell membrane that interfere with the agent’s ability to bind bacterial targets.[80] Decreased susceptibility to tigecycline has been associated with the overexpression of the AdeABC multidrug efflux pump, which confers resistance to various classes of antibiotics.[81]

Resistance to flouroquinolones is mediated by DNA topoisomerase mutations and to other classes by acquisition of mobile genetic elements or via efflux pumps [Table 3]. The mechanism involving modifications of lipopolysaccharides is also seen in the resistance of *A. baumannii* to quinolone agents from mutations in both gyrA and parC topoisomerase enzymes.[31] The plasmid-mediated quinolone resistance gene, qnrA, which encodes for the Qnr protein that protects DNA from quinolone binding, has not yet been detected in *A. baumannii*, although it has been found in other gram-negative bacteria such as the Enterobacter and Klebsiella species.[82,83]

Multiple mechanisms often work in concert to produce the same phenotype.[15,51] In a study of an epidemic MDR *Acinetobacter* strain in France, a large genomic ‘resistance island’ containing 45 resistance genes that appeared to have been acquired from Pseudomonas, Salmonella, or the Escherichia genera has been found.[33]

## EMERGENCE AND PREVALENCE OF MDR *ACINETOBACTER* SPECIES

Due to long-term evolutionary exposure to soil organisms that produce antibiotics, *Acinetobacter* sp. can develop antibiotic resistance extremely rapidly. This is in contrast to other clinical bacteria, which require greater time to acquire resistance, usually in response to therapeutic strategies. Conjugation, plasmids, and transposons (in conjunction with integrons) play an important role in the transfer of resistance determinants between different strains. Most reported cases of indigenous transmissible antibiotic resistance from *Acinetobacter* spp. have been associated with plasmids belonging to broad-host-range incompatibility groups.[7,23] The emergence of antimicrobial-resistant *Acinetobacter* species is due both to the selective pressure exerted by the use of broad-spectrum antimicrobials and transmission of strains among patients, although the relative contributions of these mechanisms are not yet known.[33]

In a surveillance study of the antibiotic susceptibility patterns of the isolates from the ICUs of five European countries (1999), the prevalence of resistance in *Acinetobacter* spp. to gentamicin was 0 – 81%, amikacin 10 – 51%, ciprofloxacin 19 – 81%, ceftazidime 0 – 81%, piperacillin-tazobactam 36 – 75%, and imipenem 5 – 19%.[84] The MYSTIC (Meropenem Yearly Susceptibility Test Information Collection) program reported the antimicrobial susceptibility of 490 *A. baumannii* strains collected in 37 centers in 11 European countries from 1997 to 2000.[85] Imipenem and meropenem were found as the most active agents against *A. baumannii*, with resistance rates of 16 and 18%, respectively. However, susceptibility testing with ampicillin / sulbactam and colistin was not performed. Subsequent data from 40 centers in 12 countries participating in the MYSTIC program (2006) revealed a substantial increase in resistance rates for meropenem (43.4%) and imipenem (42.5%).[86]

Data of the antibiotic susceptibilities of *Acinetobacter* from different geographical regions revealed that the resistance of *Acinetobacter* spp. to imipenem was in the range of no resistance to 40% (2000 – 2004).[87] In a report from a Teaching Hospital in Spain (2002), the prevalence of imipenem-resistant *Acinetobacter* spp. had increased from no resistance in 1991 to 50% in 2001.[22] Among *Acinetobacter* spp. derived from 30 European centers from the worldwide collection of SENTRY from 2001 to 2004, the proportion of strains resistant to imipenem, meropenem, ampicillin/sulbactam, and polymyxin B was: 26.3, 29.6, 51.6, and 2.7%, respectively.[85,88] Gladstone *et al*. from Vellore, India (2005), reported a prevalence of 14% carbapenem-resistant *Acinetobacter* spp., isolated from tracheal aspirates (*n* = 56).[89] In Delhi, India (2006), the prevalence of carbapenem resistance in *Acinetobacter* spp. isolated from different clinical samples was found to be almost 35%.[90] In Greece, the proportion of imipenem-resistant *A. baumannii* isolates from patients hospitalized between 1996 and 2007, in tertiary care hospitals, in several regions of the country rose from no resistance to 85% (ICUs), 60% (medical wards), and 59% (surgical wards) [Greek System for Surveillance of Antimicrobial Resistance (GSSAR): http://www.mednet.gr/whonet/]. Bloodstream isolates from the same dataset exhibited even higher resistance rates [http://www.mednet.gr/whonet/]. The prevalence of imipenem resistance in *Acinetobacter baumannii* isolated from a burns unit of USA was found to be as high as 87% (2007).[91] The above-mentioned data suggests that an antibiotic therapy should always be guided by *in vitro* susceptibility profile of the organism.

Often colistin or tigecycline are the only available treatments for MDR *A. baumannii* infections. Unfortunately, resistance to colistin has recently emerged in Europe. The European arm of the SENTRY surveillance program identified 2.7% of polymyxin B-resistant *A. baumannii* isolates collected during 2001 – 2004.[88] In a recent surveillance study from Greece, among 100 *A. baumannii* strains derived from ICU patients, 3% were colistin-resistant, whereas, the minimum inhibitory concentration (MIC) levels of tigecycline ranged between 0.12 μg/ml and 4 μg/ml.[92] Sporadic cases of infections caused by colistin-resistant isolates have been increasingly reported from Greece.[40,93] A surveillance study performed in 34 centers across UK, during 2000, reported a 2% resistance rate to colistin among 443 *A. baumannii* tested, while tigecycline MICs ranged from < 0.032 μg/ml to 16 μg/ml.[94] Sporadic strains exhibiting colistin resistance have also been reported in Slovakia.[95]

*In vitro* activity of tigecycline against MDR strains of *A. baumannii* showed promising results, but the emergence of resistance during treatment in this species has been reported.[91,96] In a recent surveillance study from Germany, tigecycline resistance among 215 *A. baumannii* was 6%, whereas, colistin resistance was 2.8%.[97]

Alarmingly high resistance rates to tigecycline (25%) have recently been reported from Turkey, but resistance of *Acinetobacter* to tigecycline should be interpreted and reported cautiously.[98]

## CLINICAL IMPLICATIONS

Although there are cases of community acquired infections caused by *Acinetobacter* spp., the primary pathogenic role of these bacteria is undoubtedly as a nosocomial pathogen.[1,6–12] Healthcare-associated pneumonia, particularly ventilator-associated pneumonia in patients confined to hospital ICUs is the most common infection caused by this organism. However, infections including bacteremia, urinary tract infection, secondary meningitis, skin and soft tissue infections, and bone infections have also been increasingly reported. Such infections are often extremely difficult to treat because of wide spread resistance of this organism to a major group of antibiotics[7,22,23] The therapeutic difficulties are coupled with the fact that these bacteria have a significant capacity for long-term survival in the hospital environment, with corresponding enhanced opportunities for transmission between patients, either via human reservoirs or via inanimate materials[7,32,33].

The incidence of severe infection caused by MDR and PDR *A. baumannii* has been increasing worldwide. Crude mortality rates of 30 – 75% have been reported for nosocomial pneumonia caused by *A. baumannii*. However, it has also been seen that mortality resulting from *A. baumannii* infection relates to the underlying cardiopulmonary and immune status of the host rather than the inherent virulence of the organism. Patients who are very ill with multisystem disease have higher mortality and morbidity rates, which may be due to their underlying illness rather than the superimposed infection with *Acinetobacter*.[99]

*Acinetobacter* spp. has been implicated as the cause of serious infections such as ventilator-associated pneumonia (VAP), urinary tract infection, endocarditis, wound infection, nosocomial meningitis, and septicaemia, mostly involving patients with impaired host defenses. However, the true frequency of nosocomial infection caused by *Acinetobacter* spp. is difficult to assess because its isolation in clinical specimens may reflect colonization rather than infection. Some clinicians believe that the recovery of *A. baumannii* in a hospitalized patient is an indicator of the severity of the underlying illness.[87] According to the SENTRY antimicrobial resistance surveillance program *Acinetobacter* spp. was among the 10 most frequently isolated pathogens causing bloodstream infections in 14 European countries participating in the program from 1997 – 2002.[100]

A systematic review of matched case control and cohort studies examining the mortality attributable to infection with or acquisition of *A. baumannii* (infection or colonization) suggested that infection with or acquisition of *A. baumannii* seemed to be associated with increased mortality. The mortality attributable to *A. baumannii* infection was found to range from 7.8 – 43%, with higher levels in patients admitted to ICUs (10 – 43%) as compared to those admitted to wards (7.8 – 23%).[101,102] With respect to morbidity, several studies have shown that *Acinetobacter* pneumonia increases the ICU stay by several days. The median length of stay with such an infection is 21 days as compared to 14 days for controls. Such an event in addition to causing inconvenience to patients puts extra financial burden on the healthcare system.[99,101]

## THERAPEUTIC OPTIONS

Historically, carbapenems have resulted in the best therapeutic response for infections caused by MDR A *baumannii*.[1] For carbapenem-resistant *A baumannii* (XDR *Acinetobacter* spp.), tigecycline and colistimethate are two of the most frequently used alternative agents [Figure 1]. The global spread of XDR *Acinetobacter* spp. is a major challenge for the healthcare industry and other drugs such as colistin and polymyxin B, and newer drugs such as tigecycline and doripenem, have been tried for treating such infections. With the emergence of PDR *Acinetobacter* spp. and the paucity of newer antimicrobial compounds, combination therapies like imipenem + ampicillin-sulbactam, rifampin + colistin, and so on, have been tried worldwide. Such regimens are not only more expensive, but the side effects and toxicity are more and the efficacy less.[87,102,103] Increasing antimicrobial resistance leaves few therapeutic options and there are no well-designed clinical trials to compare treatment regimens for MDR, XDR, and PDR *Acinetobacter* spp. infections.

Treatment of *A. baumannii* infection typically includes aminoglycosides, such as amikacin, in combination with a beta-lactamase-stable beta-lactam such as piperacillin (often along with beta-lactamase inhibitor – tazaobactam) or imipenem. Beta-lactamase inhibitors, particularly sulbactam, have intrinsic activity against many *Acinetobacter* strains. The presence of a beta-lactam agent (e.g., ampicillin) in combination with the beta-lactamase inhibitor does not appear to contribute activity or synergy.[105,106] Monotherapy with sulbactam is not recommended for severe *Acinetobacter* infection. However, Wood *et al*. reported the successful use of sulbactam to treat 14 patients with multidrug-resistant *Acinetobacter* ventilator-associated pneumonia, finding no difference in clinical outcomes between sulbactam-treated patients and 63 patients who received imipenem.[107] Levin *et al*. reported a cure rate of 67% using ampicillin-sulbactam to treat carbapenem-resistant *Acinetobacter* infection, but good patient outcomes were associated with a lower severity of illness.[108] The results of antimicrobial susceptibility tests (e.g., with agar dilution or the Etest) of beta-lactam / beta–lactamase combinations at fixed concentrations must be interpreted with caution, because they may indicate susceptibility when an isolate is actually resistant.[106]

Aminoglycoside agents, such as tobramycin and amikacin, are therapeutic options for infection with drug-resistant *Acinetobacter* isolates that retain susceptibility. These agents are usually used in conjunction with another active antimicrobial agent. Many resistant *Acinetobacter* isolates retain intermediate susceptibility to amikacin or tobramycin.

### Treatment of MDR *Acinetobacter* species

Carbapenems remain the treatment of choice if isolates retain susceptibility to this antimicrobial class. The MYSTIC surveillance program has documented discordance that favors imipenem as the more potent agent, compared to meropenem, for treatment of MDR *Acinetobacter* infection.[33] Efflux pumps may affect meropenem to a greater degree, whereas, specific beta-lactamases hydrolyze imipenem more efficiently.[33] Susceptibility testing of imipenem does not predict susceptibility to meropenem or vice versa.[33]

### Treatment of XDR and PDR *Acinetobacter* spp

With XDR *Acinetobacter* spp. infections being frequently reported, polymyxins and tigecycline should be used as the drugs of last resort for the treatment of such infections.[88,109]

Tigecycline, a new minocycline derivative, a new glycylcycline agent, received approval from the Food and Drug Administration in June 2005.[26] The drug is a parenteral, broad-spectrum, bacteriostatic agent and is approved for treatment of complicated skin and skin structure infections as well as intra-abdominal infections caused by susceptible organisms. Tigecycline has activity against the multidrug-resistant *Acinetobacter* species.[97] Tigecycline’s mechanism of action involves binding to the 30S ribosomal subunit and blocking protein synthesis. Tigecycline has a 7 to 9 L/kg volume of distribution and a half-life of approximately 42 hours. A loading dose of 100 mg is recommended, with a maintenance dose of 50 mg every 12 hours. No dose adjustment is required for patients with renal impairment or mild-to-moderate hepatic impairment. Major side effects include nausea (29.5%), vomiting (19.7%), and diarrhea (12.7%).[26]

The Clinical and Laboratory Standards Institute did not provide an interpretation of ‘susceptible’, ‘intermediate,’ and ‘resistant’ for susceptibility to tigecycline because of a lack of correlating clinical data. Without an interpretation, only the size of the area of growth inhibition could be reported. Hence, the interpretation of these findings was left up to the individual physicians. In one study, in susceptibility testing, the minimum inhibitory concentration required to inhibit a growth of 90% of organisms *in vitro* was 2.0 μg/mL for 739 isolates of *Acinetobacter*, indicating the potential clinical effectiveness of tigecycline.[26]

High-level resistance to tigecycline has been detected among some MDR *Acinetobacter* isolates and there is concern that the organism can rapidly evade this antimicrobial agent by upregulating chromosomally mediated efflux pumps.[33,52] Studies have documented overexpression of a multidrug efflux pump in *Acinetobacter* isolates with decreased susceptibility to tigecycline.[33] Given these findings and concerns about whether adequate peak serum concentrations can be achieved, tigecycline is best reserved for salvage therapy, with administration determined in consultation with an infectious diseases specialist.[33]

Combination therapy with tigecycline and other antimicrobial agents has been studied.[110] Considering all antimicrobials in combination with tigecycline, a chequerboard analysis showed 5.9% synergy, 85.7% indifference, and 8.3% antagonism.[110] Tigecycline showed synergism with levofloxacin, amikacin, imipenem, and colistin. Antagonism was observed for the tigecycline / piperacillin-tazobactam combination. Synergism was detected only among tigecycline non-susceptible strains. Time-kill assays confirmed the synergistic interaction between tigecycline and levofloxacin, amikacin, imipenem, and colistin. No antagonism was confirmed by time-kill assays.

Given the limited therapeutic options, clinicians have returned to the use of polymyxin B or polymyxin E (colistin) for XDR *Acinetobacter* infections.[33] Colistimethate is an antimicrobial produced by Bacillus colistinus. It had become commercially available in 1959. It is approved by the Food and Drug Administration for treatment of acute or chronic infections due to susceptible gram-negative bacteria. Colistimethate is hydrolyzed to colistin. Colistin acts as a cationic detergent, disturbing the bacterial cell membrane, thus increasing permeability and leading to cell death.[33] Colistin has bactericidal activity against *Acinetobacter* species and its effect is concentration-dependent. Colistin is eliminated via the kidneys and has a half-life of 1.5 to 8 hours. There are inconsistencies among manufacturers regarding the recommended dosing of colistin and the units of measurement employed.[111] The most common dose of colistimethate is 2.5 mg/kg intravenously every 12 hours, for patients with normal renal function. Data suggest that the current recommended dosing regimens may lead to serum levels of colistin that are less than the minimum inhibitory concentration (MIC) for *Acinetobacter* infections.[111]

Nephrotoxic, neurotoxic, and pulmonary toxic effects are major adverse effects associated with this drug. Dosages and interval adjustments are required for patients with creatinine clearance less than 75 mL/min. Colistin is poorly removed through hemodialysis.[26] Neurological toxicity, which was apparently dose-dependent and reversible, occurred primarily in reports published before 1970. The most common manifestation of neurological toxicity was meningeal irritation.[112]

Colistimethate was used clinically because of its proven ability to treat infections caused by MDR *A. baumannii* and other MDR organisms.[26] According to The Surveillance Network, the susceptibility of *A baumannii* isolates to polymyxin B in the United States is 95.4%.[26] Many studies have reported cure rates or improvement with colistin of 57 –77% among severely ill patients with MDR *Acinetobacter* species infections, including bacteremia, pneumonia, sepsis, CNS infection, and intra-abdominal infection.[33] Although in-depth pharmacokinetic data is lacking, colistin is reported to have relatively poor lung and CSF distribution and the clinical outcomes vary for different types of infections.[113] Various studies have reported higher favorable clinical response rates (56 – 61%) for parenteral colistin treatment of MDR *Acinetobacter* species ventilator-associated pneumonia.[33]

There is insufficient evidence to draw conclusions regarding the efficacy, safety or pharmacokinetic properties of colistin for treatment of CNS infection, although it remains an important option for salvage therapy.[114] There are case reports of successful treatment of XDR *Acinetobacter* meningitis with parenteral colistin, but its efficacy for this condition remains unclear.[114–116] Several case reports and case series report the use of intraventricular or intrathecal polymyxin therapy, with or without parenteral therapy, for the treatment of gram-negative bacterial meningitis.[114,117] Using this route of colistin administration, a cure rate of 91% has been reported in patients with *Acinetobacter* meningitis.[117] A majority of patients received systemic antimicrobial therapy in addition to the local administration of polymyxin. The problem of selecting *A. baumanni* colistin-resistant strains from a colistin-heteroresistant isolate during ongoing therapy with colistin has also been highlighted *in vitro*, in a post-neurosurgical patient.[118–120] Heteroresistance (i.e., subpopulations with varying levels of resistance to colistin) has been observed among 15 of 16 colistin susceptible *Acinetobacter* isolates studied *in vitro*.[118] Serial subcultures of the isolates, in the presence of colistin, increased the proportion of colistin-resistant subpopulations. Another study with similar findings, suggested that combination therapy may be advisable to prevent the emergence of colistin resistance during monotherapy.[119]

A lack of controlled clinical trials makes it difficult to evaluate the role of synergy or combination therapy for XDR and PDR *Acinetobacter* infection. The most readily available data are from uncontrolled case series, animal models, or *in vitro* studies. Many studies describe different combinations of antimicrobials including rifampin, sulbactam, aminoglycoside agents, colistin, and carbapenems for the management of XDR and PDR *Acinetobacter* infections.[33] However, studies have found conflicting results with the same antimicrobial combinations. A study in a mouse model of XDR *Acinetobacter* pneumonia has found that the combinations of rifampin with imipenem, tobramycin or colistin were the most effective regimens.[121] However, the use of a similar combination of rifampin plus imipenem for the treatment of carbapenem-resistant *Acinetobacter* infection has been cautioned due to a high failure rate, and the emergence of rifampin resistance in 70% of the patients who were treated with this regimen has been documented.[122] Despite the demonstration of *in vitro* synergy between the combination of imipenem and amikacin, use of the combination in a guinea pig model revealed that the combination was worse than imipenem alone for the treatment of imipenem-resistant pneumonia.[123]

Most results of the combination therapy are comparable to the cure rates reported for parenteral colistin alone and the wide variety of other agents used limits the ability to draw any conclusions with regard to combination therapy. Controlled clinical studies are needed to determine whether any antimicrobial combinations translate into useful therapeutic strategies.

### Management in children

There is paucity of literature that recommends or demonstrates the use of polymyxins for treatment of children infected with XDR and PDR *Acinetobacter* spp. A case series of critically ill children who received intravenous colistimethate for treatment of infections due to XDR gram-negative bacteria has been published.[124] The dosage of colistin administered in the case series was colistimethate at a total daily dosage of 5 mg/kg [62.500 international units (IU)/kg], administered in equally divided doses every 8 hours.[124] Five out of the seven reported patients received a ten-day colistimethate treatment and the remaining two received treatment for two and 23 days, respectively. All these infections improved with intravenous colistimethate therapy. No adverse events occurred in this case series. In another retrospective study, a case series of children with burns, focused on the efficacy and safety of colistimethate treatment also revealed similar results.[125] In both case series neither nephrotoxicity nor neurotoxicity was reported in any of the cases.

## CONTROL MEASURES

Inadequate hand hygiene remains a significant factor in the transmission of this pathogen.[126] Cross-transmission of MDR *A. baumannii* occurs via direct contact from hands and gloves from healthcare professionals to patients.[26] Rational use of antimicrobials is another important aspect to delay the emergence of XDR and PDR *Acinetobacter* spp. This can be achieved using an effective antimicrobial stewardship program having at least three components, namely, placing antibiotic policy, education regarding the stewardship program, and monitoring of the program [Figure 1]. Various infection control measures that can be adopted during routine care and during outbreak situations are summarized in Table 4. Involvement at all levels of healthcare personnel, including top management personnel, is imperative for effective implementation and success of the program.

**Table 4:** Infection control practices for patient care units

To be followed all the times
Standard precautions – including hand hygiene measures and monitoring of its compliance Contact barrier precautions Environmental cleaning and disinfection protocols Surveillance – Passive as well as Active surveillance Antimicrobial stewardship Program – Implementation and monitoring Regular training programs on infection control policies and procedures
**In outbreak situations**
Re-enforcement of above infection control practices Point source control (if source could be identified) Cohorting of patients Cohorting of health care personnel (dedicated and designated staff for designated patients) Clinical unit closure (In case the outbreak could not be managed by above measures)

## CONCLUSIONS

*Acinetobacter* spp. are rapidly spreading with emergence of extended resistance to even newer antimicrobials. They have the ability to acquire resistance at a much faster pace than other gram-negative organisms. Due to their ease of survival in the hospital environment, they have immense potential to cause nosocomial outbreaks. In addition to antibiotic resistance, their biofilm forming ability plays a crucial role in their *in-vitro* and *in-vivo* survival. Thus, to decrease the spread of *Acinetobacter* infections and reduce the pace of emergence of resistance in MDR *Acinetobacter*, it is important to promote the rational use of antimicrobials, with implementation and monitoring of the Antibiotics Stewardship Program in hospitals. Hand hygiene and barrier nursing are important to keep the spread of infection in check.

## References

[1] Fournier PE, Richet H (2006). The epidemiology and control of *Acinetobacter baumannii* in health care facilities. Clin Infect Dis.

[2] Jawad A, Heritage J, Snelling AM, Gascoyne-Binzi DM, Hawkey PM (1996). Influence of relative humidity and suspending menstrua on survival of *Acinetobacter* spp. on dry surfaces. J Clin Microbiol.

[3] Beijerinck M (1911). Pigmenten als oxydatieproducten gevormd door bacterien. Vers Konin Akad Wet Ams.

[4] Bouvet PJ, Grimont PA (1986). Taxonomy of the genus *Acinetobacter* with the recognition of *Acinetobacter baumannii* sp. nov, *Acinetobacter* haemolyticus sp. nov, *Acinetobacter* johnsonii sp. nov and *Acinetobacter* junii sp. nov and emended description of *Acinetobacter* calcoaceticus and *Acinetobacter* lwoffii. Int J Syst Bacteriol.

[5] Gerner-Smidt P (1992). Ribotyping of the *Acinetobacter* calcoaceticus-*Acinetobacter baumannii* complex. J Clin Microbiol.

[6] Gerner-Smidt P, Tjernberg I, Ursing J (1991). Reliability of phenotypic tests for identification of *Acinetobacter* species. J Clin Microbiol.

[7] Bergogne-Bérézin E, Towner KJ (1996). *Acinetobacter* spp. as nosocomial pathogens: microbiological, clinical, and epidemiological features. Clin Microbiol Rev.

[8] Lessel EF (1971). Minutes of the Subcommittee on the Taxonomy of Moraxella and Allied Bacteria. Int J Syst Bacteriol.

[9] Bouvet PJ, Jeanjean S (1989). Delineation of new proteolytic genomic species in the genus *Acinetobacter*. Res Microbiol.

[10] Tjernberg I, Ursing J (1989). Clinical strains of *Acinetobacter* classified by DNA-DNA hybridization. APMIS.

[11] Falagas ME, Koletsi PK, Bliziotis IA (2006). The diversity of definitions of multidrug-resistant (MDR) and pandrug-resistant (PDR) *Acinetobacter baumannii* and Pseudomonas aeruginosa. J Med Microbiol.

[12] Falagas ME, Karageorgopoulos DE (2008). Pandrug resistance (PDR), extensive drug resistance (XDR), and multidrug resistance (MDR) among Gram-negative bacilli: need for international harmonization in terminology. Clin Infect Dis.

[13] Simor AE, Lee M, Vearncombe M, Jones-Paul L, Barry C, Gomez M (2002). An outbreak due to multiresistant *Acinetobacter baumannii* in a burn unit: risk factors for acquisition and management. Infect Control Hosp Epidemiol.

[14] Gusten WM, Hansen EA, Cunha BA (2002). *Acinetobacter baumannii* pseudomeningitis. Heart Lung.

[15] Peleg AY, Seifert H, Paterson DL (2008). *Acinetobacter baumannii*: emergence of a successful pathogen. Clin Microbiol Rev.

[16] La Scola B, Raoult D (2004). *Acinetobacter baumannii* in human body louse.*Acinetobacter baumannii* in human body louse.

[17] Seifert H, Dijkshoorn L, Gerner-Smidt P, Pelzer N, Tjernberg I, Vaneechoutte M (1997). Distribution of *Acinetobacter* species on human skin: comparison of phenotypic and genotypic identification methods. J Clin Microbiol.

[18] Berlau J, Aucken H, Malnick H, Pitt T (1999). Distribution of *Acinetobacter* species on skin of healthy humans. Eur J Clin Microbiol Infect Dis.

[19] Dijkshoorn L, van Aken E, Shunburne L, van der Reijden TJ, Bernards AT, Nemec A (2005). Prevalence of *Acinetobacter baumannii* and other *Acinetobacter* spp. in faecal samples from non-hospitalised individuals. Clin Microbiol Infect.

[20] Somerville DA, Noble WC (1970). A note on the gram negative bacilli of human skin. Rev Eur Etud Clin Biol.

[21] Taplin D, Zaias N (1963). The human skin as a source of mima-herellea infections. JAMA.

[22] Cisneros JM, Rodríguez-Baño J (2002). Nosocomial bacteremia due to *Acinetobacter baumannii*: epidemiology, clinical features and treatment. Clin Microbiol Infect.

[23] Hartzell JD, Kim AS, Kortepeter MG, Moran KA (2007). *Acinetobacter* pneumonia: a review. MedGenMed.

[24] Allen KD, Green HT (1987). Hospital outbreak of multi-resistant *Acinetobacter* anitratus: an airborne mode of spread?. J Hosp Infect.

[25] Buxton AE, Anderson RL, Werdegar D, Atlas E (1978). Nosocomial respiratory tract infection and colonization with *Acinetobacter* calcoaceticus. Epidemiologic characteristics. Am J Med.

[26] Montefour K, Frieden J, Hurst S, Helmich C, Headley D, Martin M (2008). *Acinetobacter baumannii*: an emerging multidrug-resistant pathogen in critical care. Crit Care Nurse.

[27] Falagas ME, Karveli EA (2007). The changing global epidemiology of *Acinetobacter baumannii* infections: a development with major public health implications. Clin Microbiol Infect.

[28] Bernards AT, Harinck HI, Dijkshoorn L, van der Reijden TJ, van den Broek PJ (2004). Persistent *Acinetobacter baumannii*? Look inside your medical equipment. Infect Control Hosp Epidemiol.

[29] Das I, Lambert P, Hill D, Noy M, Bion J, Elliott T (2002). Carbapenem-resistant *Acinetobacter* and role of curtains in an outbreak in intensive care units. J Hosp Infect.

[30] Podnos YD, Cinat ME, Wilson SE, Cooke J, Gornick W, Thrupp LD (2001). Eradication of multidrug resistant *Acinetobacter* from an intensive care unit. Surg Infect (Larchmt)).

[31] Rello J, Diaz E (2003). *Acinetobacter baumannii*: a threat for the ICU?. Intensive Care Med.

[32] Jawad A, Seifert H, Snelling AM, Heritage J, Hawkey PM (1998). Survival of *Acinetobacter baumannii* on dry surfaces: comparison of outbreak and sporadic isolates. J Clin Microbiol.

[33] Maragakis LL, Perl TM (2008). *Acinetobacter baumannii*: epidemiology, antimicrobial resistance, and treatment options. Clin Infect Dis.

[34] Playford EG, Craig JC, Iredell JR (2007). Carbapenem-resistant *Acinetobacter baumannii* in intensive care unit patients: risk factors for acquisition, infection and their consequences. J Hosp Infect.

[35] Falagas ME, Kopterides P (2006). Risk factors for the isolation of multidrug-resistant *Acinetobacter baumannii* and Pseudomonas aeruginosa: a systematic review of the literature. J Hosp Infect.

[36] Landman D, Quale JM, Mayorga D, Adedeji A, Vangala K, Ravishankar J (2002). Citywide clonal outbreak of multiresistant *Acinetobacter baumannii* and Pseudomonas aeruginosa in Brooklyn, NY: the preantibiotic era has returned. Arch Intern Med.

[37] Cisneros JM, Rodríguez-Baño J, Fernández-Cuenca F, Ribera A, Vila J, Pascual A, Martínez-Martínez L (2005). Risk-factors for the acquisition of imipenem-resistant *Acinetobacter baumannii* in Spain a nationwide study. Clin Microbiol Infect.

[38] Medina J, Formento C, Pontet J, Curbelo A, Bazet C, Gerez J (2007). Prospective study of risk factors for ventilator-associated pneumonia caused by *Acinetobacter* species. J Crit Care.

[39] Katsaragakis S, Markogiannakis H, Toutouzas KG, Drimousis P, Larentzakis A, Theodoraki EM (2008). *Acinetobacter baumannii* infections in a surgical intensive care unit: predictors of multidrug resistance. World J Surg.

[40] Matthaiou DK, Michalopoulos A, Rafailidis PI, Karageorgopoulos DE, Papaioannou V, Ntani G (2008). Risk factors associated with the isolation of colistin-resistant gram-negative bacteria: a matched case-control study. Crit Care Med.

[41] Sunenshine RH, Wright MO, Maragakis LL, Harris AD, Song X, Hebden J (2007). Multidrug-resistant *Acinetobacter* infection mortality rate and length of hospitalization. Emerg Infect Dis.

[42] Marchaim D, Navon-Venezia S, Leavitt A, Chmelnitsky I, Schwaber MJ, Carmeli Y (2007). Molecular and epidemiologic study of polyclonal outbreaks of multidrug-resistant *Acinetobacter baumannii* infection in an Israeli hospital. Infect Control Hosp Epidemiol.

[43] Oteo J, García-Estébanez C, Migueláñez S, Campos J, Martí S, Vila J, Domínguez MA (2007). Genotypic diversity of imipenem resistant isolates of *Acinetobacter baumannii* in Spain. J Infect.

[44] Bano JR, Marti S, Soto S, Cuenca FF, Cisneros JM, Pachon J (2008). Biofilm Formation In *Acinetobacter baumannii*: Associated Features And Clinical Implications. Clin Microbiol Infect.

[45] Lee HW, Koh YM, Kim J, Lee JC, Lee YC, Seol SY (2008). Capacity of multidrug-resistant clinical isolates of *Acinetobacter baumannii* to form biofilm and adhere to epithelial cell surfaces. Clin Microbiol Infect.

[46] Canduela MJ, Gallego L, Sevillano E, Valderrey C, Calvo F, Pérez J (2006). Evolution of multidrug-resistant *Acinetobacter baumannii* isolates obtained from elderly patients with respiratory tract infections. J Antimicrob Chemother.

[47] Zeana C, Larson E, Sahni J, Bayuga SJ, Wu F, Della-Latta P (2003). The epidemiology of multidrug-resistant *Acinetobacter baumannii*: does the community represent a reservoir?. Infect Control Hosp Epidemiol.

[48] Villers D, Espaze E, Coste-Burel M, Giauffret F, Ninin E, Nicolas F (1998). Nosocomial *Acinetobacter baumannii* infections: microbiological and clinical epidemiology. Ann Intern Med.

[49] Bergogne-Bérézin E (1995). The increasing significance of outbreaks of *Acinetobacter* spp.: the need for control and new agents. J Hosp Infect.

[50] Go ES, Urban C, Burns J, Kreiswirth B, Eisner W, Mariano N (1994). Clinical and molecular epidemiology of *Acinetobacter* infections sensitive only to polymyxin B and sulbactam. Lancet.

[51] Fernández-Cuenca F, Martínez-Martínez L, Conejo MC, Ayala JA, Perea EJ, Pascual A (2003). Relationship between beta-lactamase production, outer membrane protein and penicillin-binding protein profiles on the activity of carbapenems against clinical isolates of *Acinetobacter baumannii*. J Antimicrob Chemother.

[52] Rice LB (2006). Challenges in identifying new antimicrobial agents effective for treating infections with *Acinetobacter baumannii* and Pseudomonas aeruginosa. Clin Infect Dis.

[53] Hujer KM, Hamza NS, Hujer AM, Perez F, Helfand MS, Bethel CR (2005). Identification of a new allelic variant of the *Acinetobacter baumannii* cephalosporinase, ADC-7 beta-lactamase: defining a unique family of class C enzymes. Antimicrob Agents Chemother.

[54] Perilli M, Felici A, Oratore A, Cornaglia G, Bonfiglio G, Rossolini GM (1996). Characterization of the chromosomal cephalosporinases produced by *Acinetobacter* lwoffii and *Acinetobacter baumannii* clinical isolates. Antimicrob Agents Chemother.

[55] Hujer KM, Hujer AM, Hulten EA, Bajaksouzian S, Adams JM, Donskey CJ (2006). Analysis of antibiotic resistance genes in multidrug-resistant *Acinetobacter* sp. isolates from military and civilian patients treated at the Walter Reed Army Medical Center. Antimicrob Agents Chemother.

[56] Ruiz M, Marti S, Fernandez-Cuenca F, Pascual A, Vila J (2007). Prevalence of IS(Aba1) in epidemiologically unrelated *Acinetobacter baumannii* clinical isolates. FEMS Microbiol Lett.

[57] Bou G, Martínez-Beltrán J (2000). Cloning, nucleotide sequencing, and analysis of the gene encoding an AmpC beta-lactamase in *Acinetobacter baumannii*. Antimicrob Agents Chemother.

[58] Héritier C, Poirel L, Nordmann P (2006). Cephalosporinase over-expression resulting from insertion of ISAba1 in *Acinetobacter baumannii*. Clin Microbiol Infect.

[59] Corvec S, Caroff N, Espaze E, Giraudeau C, Drugeon H, Reynaud A (2003). AmpC cephalosporinase hyperproduction in *Acinetobacter baumannii* clinical strains. J Antimicrob Chemother.

[60] Segal H, Nelson EC, Elisha BG (2004). Genetic environment and transcription of ampC in an *Acinetobacter baumannii* clinical isolate. Antimicrob Agents Chemother.

[61] Bonomo RA, Szabo D (2006). Mechanisms of multidrug resistance in *Acinetobacter* species and Pseudomonas aeruginosa. Clin Infect Dis.

[62] Héritier C, Poirel L, Lambert T, Nordmann P (2005). Contribution of acquired carbapenem-hydrolyzing oxacillinases to carbapenem resistance in *Acinetobacter baumannii*. Antimicrob Agents Chemother.

[63] Afzal-Shah M, Woodford N, Livermore DM (2001). Characterization of OXA-25, OXA-26, and OXA-27, molecular class D beta-lactamases associated with carbapenem resistance in clinical isolates of *Acinetobacter baumannii*. Antimicrob Agents Chemother.

[64] Brown S, Amyes S (2006). OXA (beta)-lactamases in *Acinetobacter*: the story so far. J Antimicrob Chemother.

[65] Poirel L, Pitout JD, Nordmann P (2007). Carbapenemases: molecular diversity and clinical consequences. Future Microbiol.

[66] Walther-Rasmussen J, Høiby N (2006). OXA-type carbapenemases. J Antimicrob Chemother.

[67] Poirel L, Nordmann P (2006). Genetic structures at the origin of acquisition and expression of the carbapenem-hydrolyzing oxacillinase gene blaOXA-58 in *Acinetobacter baumannii*. Antimicrob Agents Chemother.

[68] Coelho JM, Turton JF, Kaufmann ME, Glover J, Woodford N, Warner M (2006). Occurrence of carbapenem-resistant *Acinetobacter baumannii* clones at multiple hospitals in London and Southeast England. J Clin Microbiol.

[69] Suárez CJ, Lolans K, Villegas MV, Quinn JP (2005). Mechanisms of resistance to beta-lactams in some common Gram-negative bacteria causing nosocomial infections. ._Expert Rev Anti Infect Ther.

[70] Héritier C, Poirel L, Fournier PE, Claverie JM, Raoult D, Nordmann P (2005). Characterization of the naturally occurring oxacillinase of *Acinetobacter baumannii*. Antimicrob Agents Chemother.

[71] Livermore DM, Woodford N (2006). The beta-lactamase threat in Enterobacteriaceae, Pseudomonas and *Acinetobacter*. Trends Microbiol.

[72] Walsh TR, Toleman MA, Poirel L, Nordmann P (2005). Metallo-beta-lactamases: the quiet before the storm?. Clin Microbiol Rev.

[73] Vahaboglu H, Oztürk R, Aygün G, Coşkunkan F, Yaman A, Kaygusuz A (1997). Widespread detection of PER-1-type extended-spectrum beta-lactamases among nosocomial *Acinetobacter* and Pseudomonas aeruginosa isolates in Turkey: a nationwide multicenter study. Widespread detection of PER-1-type extended-spectrum beta-lactamases among nosocomial *Acinetobacter* and Pseudomonas aeruginosa isolates in Turkey: a nationwide multicenter study.

[74] Poirel L, Cabanne L, Vahaboglu H, Nordmann P (2005). Genetic environment and expression of the extended-spectrum beta-lactamase blaPER-1 gene in gram-negative bacteria. Antimicrob Agents Chemother.

[75] Poirel L, Menuteau O, Agoli N, Cattoen C, Nordmann P (2003). Outbreak of extended-spectrum beta-lactamase VEB-1-producing isolates of *Acinetobacter baumannii* in a French hospital. J Clin Microbiol.

[76] Girlich D, Naas T, Leelaporn A, Poirel L, Fennewald M, Nordmann P (2002). Nosocomial spread of the integron-located veb-1-like cassette encoding an extended-pectrum beta-lactamase in Pseudomonas aeruginosa in Thailand. Clin Infect Dis.

[77] Queenan AM, Bush K (2007). Carbapenemases: the versatile beta-lactamases. Clin Microbiol Rev.

[78] Seward RJ, Lambert T, Towner KJ (1998). Molecular epidemiology of aminoglycoside resistance in *Acinetobacter* spp. J Med Microbiol.

[79] Nemec A, Dolzani L, Brisse S, van den Broek P, Dijkshoorn L (2004). Diversity of aminoglycoside-resistance genes and their association with class 1 integrons among strains of pan-European *Acinetobacter baumannii* clones. J Med Microbiol.

[80] Li J, Nation RL, Milne RW, Turnidge JD, Coulthard K (2005). Evaluation of colistin as an agent against multi-resistant Gram-negative bacteria. Int J Antimicrob Agents.

[81] Giamarellou H, Antoniadou A, Kanellakopoulou K (2008). *Acinetobacter baumannii*: a universal threat to public health?. Int J Antimicrob Agents.

[82] Vila J, Ruiz J, Goñi P, Marcos A, Jimenez de Anta T (1995). Mutation in the gyrA gene of quinolone-resistant clinical isolates of *Acinetobacter baumannii*. Antimicrob Agents Chemother.

[83] Vila J, Ruiz J, Goñi P, Jimenez de Anta T (1997). Quinolone-resistance mutations in the topoisomerase IV parC gene of *Acinetobacter baumannii*. J Antimicrob Chemother.

[84] Hanberger H, Garcia-Rodriguez JA, Gobernado M, Goossens H, Nilsson LE, Struelens MJ (1999). Antibiotic susceptibility among aerobic gram-negative bacilli in intensive care units in 5 European countries. French and Portuguese ICU Study Groups. JAMA.

[85] Turner PJ, Greenhalgh JM (2003). MYSTIC Study Group (Europe) The activity of meropenem and comparators against *Acinetobacter* strains isolated from European hospitals, 1997-2000. Clin Microbiol Infect.

[86] Turner PJ (2008). Meropenem activity against European isolates: report on the MYSTIC (Meropenem Yearly Susceptibility Test Information Collection) 2006 results. Diagn Microbiol Infect Dis.

[87] Perez F, Hujer AM, Hujer KM, Decker BK, Rather PN, Bonomo RA (2007). Global challenge of multidrug-resistant *Acinetobacter baumannii*. Antimicrob Agents Chemother.

[88] Gales AC, Jones RN, Sader HS (2006). Global assessment of the antimicrobial activity of polymyxin B against 54 731 clinical isolates of Gram-negative bacilli: report from the SENTRY antimicrobial surveillance programme (2001-2004). Clin Microbiol Infect.

[89] Gladstone P, Rajendran P, Brahmadathan KN (2005). Incidence of carbapenem resistant nonfermenting gram negative bacilli from patients with respiratory infections in the intensive care units. Indian J Med Microbiol.

[90] Sinha M, Srinivasa H, Macaden R (2007). Antibiotic resistance profile & extended spectrum beta-lactamase (ESBL) production in *Acinetobacter* species. Indian J Med Res.

[91] Trottier V, Segura PG, Namias N, King D, Pizano LR, Schulman CI (2007). Outcomes of *Acinetobacter baumannii* infection in critically ill burned patients. J Burn Care Res.

[92] Souli M, Kontopidou FV, Koratzanis E, Antoniadou A, Giannitsioti E, Evangelopoulou P (2006). *in vitro* activity of tigecycline against multiple-drug-resistant, including pan-resistant, gram-negative and gram-positive clinical isolates from Greek hospitals. Antimicrob Agents Chemother.

[93] Falagas ME, Rafailidis PI, Matthaiou DK, Virtzili S, Nikita D, Michalopoulos A (2008). Pandrug-resistant Klebsiella pneumoniae, Pseudomonas aeruginosa and *Acinetobacter baumannii* infections: characteristics and outcome in a series of 28 patients. Int J Antimicrob Agents.

[94] Henwood CJ, Gatward T, Warner M, James D, Stockdale MW, Spence RP (2002). Antibiotic resistance among clinical isolates of *Acinetobacter* in the UK, and *in vitro* evaluation of tigecycline (GAR-936). J Antimicrob Chemother.

[95] Beno P, Krcmery V, Demitrovicova A (2006). Bacteraemia in cancer patients caused by colistin-resistant Gram-negative bacilli after previous exposure to ciprofloxacin and/or colistin. Clin Microbiol Infect.

[96] Rodloff AC, Leclercq R, Debbia EA, Cantón R, Oppenheim BA, Dowzicky MJ (2008). Comparative analysis of antimicrobial susceptibility among organisms from France, Germany, Italy, Spain and the UK as part of the tigecycline evaluation and surveillance trial. Clin Microbiol Infect.

[97] Seifert H, Stefanik D, Wisplinghoff H (2006). Comparative *in vitro* activities of tigecycline and 11 other antimicrobial agents against 215 epidemiologically defined multidrug-resistant *Acinetobacter baumannii* isolates. J Antimicrob Chemother.

[98] Dizbay M, Altuncekic A, Sezer BE, Ozdemir K, Arman D (2008). Colistin and tigecycline susceptibility among multidrug-resistant *Acinetobacter baumannii* isolated from ventilator-associated pneumonia. Int J Antimicrob Agents.

[99] Chastre J, Trouillet JL, Vuagnat A, Joly-Guillou ML (1996). Nosocomial infections caused by *Acinetobacter* spp. Microbiology, Epidemiology, Infections, Management. Danvers: CRC press.

[100] Biedenbach DJ, Moet GJ, Jones RN (2004). Occurrence and antimicrobial resistance pattern comparisons among bloodstream infection isolates from the SENTRY Antimicrobial Surveillance Program (1997-2002). Diagn Microbiol Infect Dis.

[101] Fagon JY, Chastre J, Hance AJ, Montravers P, Novara A, Gibert C (1993). Nosocomial pneumonia in ventilated patients: a cohort study evaluating attributable mortality and hospital stay. Am J Med.

[102] Falagas ME, Bliziotis IA, Siempos II (2006). Attributable mortality of *Acinetobacter baumannii* infections in critically ill patients: a systematic review of matched cohort and case-control studies. Crit Care.

[103] Marques MB, Brookings ES, Moser SA, Sonke PB, Waites KB (1997). Comparative *in vitro* antimicrobial susceptibilities of nosocomial isolates of *Acinetobacter baumannii* and synergistic activities of nine antimicrobial combinations. Antimicrob Agents Chemother.

[104] Doi Y, Husain S, Potoski BA, McCurry KR, Paterson DL (2009). Extensively drug-resistant *Acinetobacter baumannii*. Emerg Infect Dis.

[105] Brauers J, Frank U, Kresken M, Rodloff AC, Seifert H (2005). Activities of various beta-lactams and beta-lactam/beta-lactamase inhibitor combinations against *Acinetobacter baumannii* and *Acinetobacter* DNA group 3 strains. Clin Microbiol Infect.

[106] Higgins PG, Wisplinghoff H, Stefanik D, Seifert H (2004). *in vitro* activities of the beta-lactamase inhibitors clavulanic acid, sulbactam, and tazobactam alone or in combination with beta-lactams against epidemiologically characterized multidrug-resistant *Acinetobacter baumannii* strains. Antimicrob Agents Chemother.

[107] Wood GC, Hanes SD, Croce MA, Fabian TC, Boucher BA (2002). Comparison of ampicillin-sulbactam and imipenem-cilastatin for the treatment of *Acinetobacter* ventilator-associated pneumonia. Clin Infect Dis.

[108] Levin AS, Levy CE, Manrique AE, Medeiros EA, Costa SF (2003). Severe nosocomial infections with imipenem-resistant *Acinetobacter baumannii* treated with ampicillin/sulbactam. Int J Antimicrob Agents.

[109] Giske CG, Monnet DL, Cars O, Carmeli Y (2008). ReAct-Action on Antibiotic Resistance. Clinical and economic impact of common multidrug-resistant gram-negative bacilli. Antimicrob Agents Chemother.

[110] Principe L, D’Arezzo S, Capone A, Petrosillo N, Visca P (2009). *in vitro* activity of tigecycline in combination with various antimicrobials against multidrug resistant *Acinetobacter baumannii*. Ann Clin Microbiol Antimicrob.

[111] Li J, Nation RL, Turnidge JD, Milne RW, Coulthard K, Rayner CR (2006). Colistin: the re-emerging antibiotic for multidrug-resistant Gram-negative bacterial infections. Lancet Infect Dis.

[112] Petrosillo N, Chinello P, Proietti MF, Cecchini L, Masala M, Franchi C (2005). Combined colistin and rifampicin therapy for carbapenem-resistant *Acinetobacter baumannii* infections: clinical outcome and adverse events. Clin Microbiol Infect.

[113] Levin AS, Barone AA, Penço J, Santos MV, Marinho IS, Arruda EA (1999). Intravenous colistin as therapy for nosocomial infections caused by multidrug-resistant Pseudomonas aeruginosa and *Acinetobacter baumannii*. Clin Infect Dis.

[114] Katragkou A, Roilides E (2005). Successful treatment of multidrug-resistant *Acinetobacter baumannii* central nervous system infections with colistin. J Clin Microbiols.

[115] Fulnecky EJ, Wright D, Scheld WM, Kanawati L, Shoham S (2005). Amikacin and colistin for treatment of *Acinetobacter baumannii* meningitis. J Infect.

[116] Kim BN, Peleg AY, Lodise TP, Lipman J, Li J, Nation R, Paterson DL (2009). Management of meningitis due to antibiotic-resistant *Acinetobacter* species. Lancet Infect Dis.

[117] Falagas ME, Bliziotis IA, Tam VH (2007). Intraventricular or intrathecal use of polymyxins in patients with Gram-negative meningitis: a systematic review of the available evidence. IInt J Antimicrob Agents.

[118] Li J, Rayner CR, Nation RL, Owen RJ, Spelman D, Tan KE (2006). Heteroresistance to colistin in multidrug-resistant *Acinetobacter baumannii*. Antimicrob Agents Chemother.

[119] Owen RJ, Li J, Nation RL, Spelman D (2007). *in vitro* pharmacodynamics of colistin against *Acinetobacter baumannii* clinical isolates. J Antimicrob Chemother.

[120] Hernan RC, Karina B, Gabriela G, Marcela N, Carlos V, Angela F (2009). Selection of colistin-resistant *Acinetobacter baumannii* isolates in postneurosurgical meningitis in an intensive care unit with high presence of heteroresistance to colistin. Diagn Microbiol Infect Dis.

[121] Montero A, Ariza J, Corbella X, Doménech A, Cabellos C, Ayats J (2004). Antibiotic combinations for serious infections caused by carbapenem-resistant *Acinetobacter baumannii* in a mouse pneumonia model. J Antimicrob Chemother.

[122] Saballs M, Pujol M, Tubau F, Peña C, Montero A, Domínguez MA (2006). Rifampicin/imipenem combination in the treatment of carbapenem-resistant *Acinetobacter baumannii* infections. J Antimicrob Chemother.

[123] Bernabeu-Wittel M, Pichardo C, García-Curiel A, Pachón-Ibáñez ME, Ibáñez-Martínez J, Jiménez-Mejías ME (2005). Pharmacokinetic/pharmacodynamic assessment of the *in-vivo* efficacy of imipenem alone or in combination with amikacin for the treatment of experimental multiresistant *Acinetobacter baumannii* pneumonia. Clin Microbiol Infect.

[124] Falagas ME, Sideri G, Vouloumanou EK, Papadatos JH, Kafetzis DA (2009). Intravenous colistimethate (colistin) use in critically ill children without cystic fibrosis. Pediatr Infect Dis J.

[125] Goverman J, Weber JM, Keaney TJ, Sheridan RL (2007). Intravenous colistin for the treatment of multidrug resistant, gram-negative infection in the pediatric burn population. J Burn Care Res.

[126] Fierobe L, Lucet JC, Decré D, Muller-Serieys C, Deleuze A, Joly-Guillou ML (2001). An outbreak of imipenem-resistant *Acinetobacter baumannii* in critically ill surgical patients. Infect Control Hosp Epidemiol.

